# Exosomes derived from embryonal and alveolar rhabdomyosarcoma carry differential miRNA cargo and promote invasion of recipient fibroblasts

**DOI:** 10.1038/srep37088

**Published:** 2016-11-17

**Authors:** Sandra E. Ghayad, Ghina Rammal, Farah Ghamloush, Hussein Basma, Rihab Nasr, Mona Diab-Assaf, Claude Chelala, Raya Saab

**Affiliations:** 1 Department of Biology, Faculty of Science II, EDST, Lebanese University, Fanar, Lebanon; 2 Department of Pediatric and Adolescent Medicine, American University of Beirut, Beirut, Lebanon; 3 Department of Anatomy, Cell Biology and Physiology, American University of Beirut, Beirut, Lebanon; 4 Department of Chemistry and Biochemistry, Faculty of Sciences II, EDST, Lebanese University, Fanar, Lebanon; 5 Centre for Molecular Oncology, Barts Cancer Institute, Barts and the London School of Medicine and Dentistry, Queen Mary University of London, London, UK

## Abstract

Rhabdomyosarcoma (RMS) is an aggressive childhood soft tissue tumor, which exists in oncoprotein PAX-FOXO1 fusion positive and fusion negative subtypes, with the fusion-positive RMS being characterized by a more aggressive clinical behavior. Exosomes are small membranous vesicles secreted into body fluids by multiple cell types, including tumor cells, and have been implicated in metastatic progression through paracrine signaling. We characterized exosomes secreted by a panel of 5 RMS cell lines. Expression array analysis showed that, for both fusion-positive and fusion-negative cells, exosome miRNA clustered well together and to a higher extent than cellular miRNA. While enriched miRNA in exosomes of fusion-negative RMS cells were distinct from those of fusion-positive RMS cells, the most significant predicted disease and functions in both groups were related to processes relevant to cancer and tissue remodelling. Functionally, we found that RMS-derived exosomes exerted a positive effect on cellular proliferation of recipient RMS cells and fibroblasts, induced cellular migration and invasion of fibroblasts, and promoted angiogenesis. These findings show that RMS-derived exosomes enhance invasive properties of recipient cells, and that exosome content of fusion-positive RMS is different than that of fusion-negative RMS, possibly contributing to the different metastatic propensity of the two subtypes.

Rhabdomyosarcoma (RMS) is an aggressive childhood soft tissue tumor thought to arise from primitive mesenchymal cells with evidence of myogenic differentiation (reviewed in ref. 1). RMS occurs as two main histologic subtypes: alveolar (ARMS) and embryonal (ERMS) histologies. The alveolar subtype is characterized, in the majority of cases, by a chromosomal translocation t(2;13) (q35;q14), resulting in the fusion of the gene encoding the DNA binding domain of Paired Box 3 (PAX3) with the gene encoding the transcriptional activation domain of Forkhead Box O1 (FOXO1, previously known as FKHR) on chromosome 13 (reviewed in ref. 1). An alternate chromosomal translocation t(1;13) (p36;q14) results in a fusion between PAX7 on chromosome 1 and FOXO1, and occurs in a minor proportion of ARMS (reviewed in ref. 2). These ARMS-specific translocations result in an oncogenic PAX3-FOXO1 or PAX7-FOXO1 fusion protein, respectively, which contribute to the aggressive and metastatic behavior of ARMS (reviewed in ref. 2). Indeed, ARMS tumors are metastatic at diagnosis in approximately 80% of patients, as compared to only 20% in ERMS, and are associated with poor outcome despite current multimodality therapy. Recently, it has been suggested that fusion status may be a better stratification marker than histology, and classification of RMS into fusion-positive versus fusion-negative (rather than ARMS and ERMS, respectively) may be more useful in prognostication and clinical allocation of therapy^3^. Better understanding of the mechanisms by which both subtypes of RMS develop metastatic properties are needed, for development of novel therapies and improvements in outcome of patients with advanced disease^4^.

Exosomes are small secreted membrane-bound particles measuring 30 to 120 nm in diameter, that have been shown to play important roles in cell-cell signaling and cellular communication, promoting secretion of growth factors, cytokines, and angiogenic factors by stromal cells, proliferation of endothelial cells, and metastasis (reviewed in ref. 5). Upon endocytosis, exosomes deliver their active components, including proteins, RNA and miRNA directly into the cytoplasm of recipient cells, and can influence their biological processes^6^. Emerging evidence indicates that packaging of miRNA into exosomes is not random and may rely on sequence-specific and secondary structure^7^,^8^. Exosomes derived from cancer cells have been demonstrated to promote angiogenesis, invasion, migration and proliferation in recipient cells to support tumor growth^9^. Some of the most compelling studies for an important role of exosomes are in the highly metastatic tumor melanoma, where transfer of protein via exosomes was shown to be responsible for preparing the metastatic niche in multiple organs, thus facilitating melanoma metastasis^10^. In pediatric cancers, few studies have investigated the role of exosomes in tumor biology. Studies reported that Ewing sarcoma, medulloblastoma, and neuroblastoma cell lines secrete exosomes, with specific identifiable cargo^11^,^12^,^13^.

RMS is a particularly interesting tumor where paracrine signaling is likely important, specifically the fusion-positive subtype, which is known to be highly metastatic. We hypothesized that RMS-derived exosomes enhance invasiveness of RMS cells and associated fibroblasts via paracrine signaling, thus contributing to the known metastatic behavior of this aggressive tumor.

## Results

### RMS cells secrete detectable amounts of exosomes

**Figure 1:**
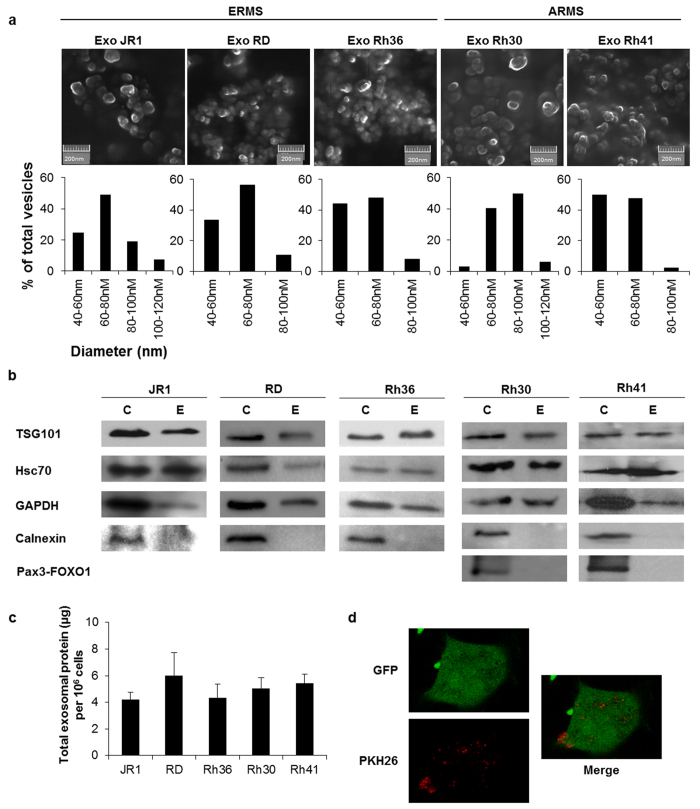
Characterization of exosomes derived from RMS cells. (**a**) Representative SEM micrographs of exosomes purified from the respective ERMS and ARMS cells. Histograms represent percentage of extracellular vesicles per diameter range. (**b**) Western blot for the indicated proteins in exosome “E” and respective cell “C” lysates, as indicated, for each of the RMS cell lines. (**c**) Histogram representing the average total exosome proteins (μg) quantified by Bradford assay per 1 million cells. Bars represent standard deviation. (**d**) Representative confocal microscopy images (single z-slices) for GFP-transduced MEFs (green) after 2 hours of treatment with PKH 26-labeled exosomes (red) derived from JR1 cells. Images are representative of 2 independent experiments.

We evaluated a panel of 5 well-characterized RMS cell lines for exosome secretion. All tested cell lines of both fusion-negative (and embryonal histology) RMS, namely the JR1, RD and Rh36 cell lines, and the fusion-positive (and alveolar histology) Rh30 and Rh41 cell lines, were found to secrete small vesicles visualized using scanning electron microscopy (Fig. 1a, upper panel). Measurement of the isolated vesicles diameter confirmed a size range of 40–120 nm (Fig. 1a, lower panel), consistent with exosomes^5^. The identity of these vesicles as exosomes was confirmed by analysis of their protein cargo, as western blotting showed that they contained the exosome protein markers TSG101, HSC70 and GAPDH (Fig. 1b)^14^, and were devoid of Calnexin (Fig. 1b), a cellular protein that is localized to the endoplasmic reticulum and therefore excluded from exosomes^15^. Notably, the fusion oncoprotein PAX3-FOXO1 was not present in exosomes derived from the fusion-positive RMS cell lines Rh30 and Rh41, despite being clearly present in the corresponding cell lysates (Fig. 1b). Exosomes collected at 72 hours of culture contained an average of 5 micrograms of protein per million cells, which was relatively uniform across all examined cell lines (Fig. 1c). To evaluate whether RMS-derived exosomes are indeed taken up by recipient cells, we used fibroblasts transduced with retrovirus expressing green fluorescent protein (GFP), and treated them with RMS-derived exosomes stained red with the PKH26 membrane dye. Confocal imaging showed that PKH26-stained exosomes were indeed taken up by cultured fibroblasts, clearly evident by 2 hours after exposure (Fig. 1d). We conclude that RMS cells secrete exosomes that can be readily isolated, and that these exosomes are taken up by recipient cells.

### RMS-derived exosomes carry specific miRNA relevant to cancer signaling networks

**Figure 2:**
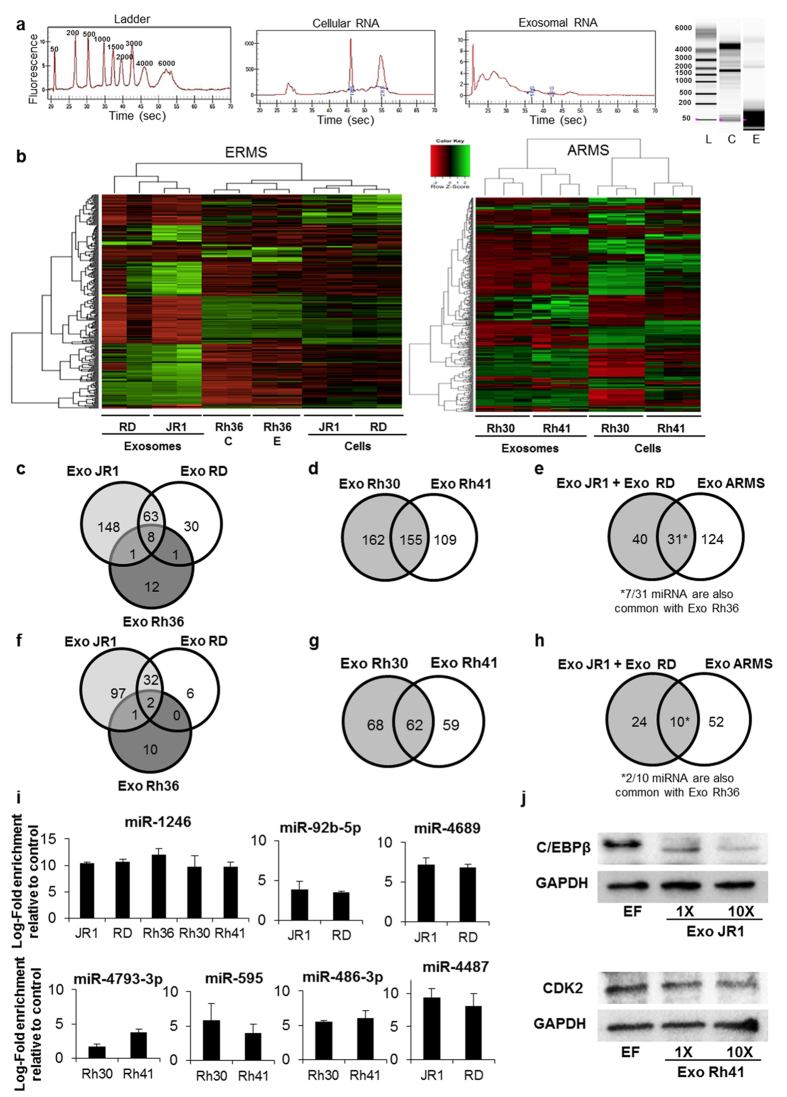
miRNA profiling of RMS exosomes compared to donor cells. (**a**) Representative RNA quality verification using the Experion electrophoresis system showing the presence of 18 S and 28 S rRNA subunits in total RNA in cells “C” and RNA in exosomes “E” compared to the ladder “L”. Numbers over peaks and next to ladder bands refer to nucleotide size. (**b**) Hierarchical clustering of all deregulated miRNA probe sets in ERMS (n = 2) and ARMS (n = 3) exosomes compared to their respective donor cells in independent cell cultures. Each column represents a cell line replicate, and each row represents a miRNA. The scaled expression of each miRNA, denoted as the row *Z-score*, is plotted in green–red colour scale. High expression levels are indicated in green and low expression levels are shown in red. (**c**–**e**) Venn diagram of all commonly deregulated (enriched and depleted) miRNA in exosomes derived from (**c**) the 3 ERMS cell lines; (**d**) the 2 ARMS cell lines and (**e**), all 4 specified cell lines excluding Rh36. (**f**–**h**) Venn diagram of only commonly enriched miRNA in exosomes derived from (**f**), the 3 ERMS cell lines; (**g**) the 2 ARMS cell lines; and (**h**) all 4 specified cell lines excluding Rh36. (**i**) Validation by qRT-PCR of the enrichment of the specified miRNA in exosomes derived from the specified RMS cells. The small RNA RNU6 was used as endogenous control. Histograms represent the mean log-fold change of miRNA in exosomes relative to respective donor cells, from at least two independent experiments each performed in triplicate. Bars represent standard deviation. (**j**) Western blot for the indicated target proteins in lysates of BJ cells after treatment with 1X and 10X JR1- or Rh41-derived exosomes for 48 hours, as specified, compared to the control exosome-free media ‘EF’.

To characterize the cargo of RMS-derived exosomes, we extracted total RNA from both exosomes and their respective cells. Consistent with other studies^13^,^16^,^17^,^18^, a preponderance of small-sized RNA was detected in RMS exosomes as compared to the respective cell lysates (Fig. 2a). We therefore decided to focus on miRNA content of RMS-derived exosomes, using the Affymetrix GeneChip miRNA 3.0 array. Unsupervised hierarchical clustering showed that, in ARMS cell lines, exosome miRNA clustered together and separately from cellular miRNA (Fig. 2b, right panel). The same was seen for ERMS cell lines JR1 and RD (Fig. 2b, left panel). The Rh36 cell line was the only one where exosome and cellular miRNA clustered together reflecting the miRNA content of donor cells (Fig. 2b, left panel), and they clustered along with the cellular miRNA of the other ERMS cell lines.

We identified a total of 220 differentially deregulated (either enriched or depleted) miRNA in JR1-derived exosomes as compared to cell lysate, 102 in RD-derived exosomes and 22 in Rh36-derived exosomes. JR1- and RD-derived exosomes shared 71 deregulated miRNA, whereas 8 miRNA were deregulated in common with Rh36-derived exosomes (Fig. 2c). For ARMS cells, we found 317 and 264 differentially deregulated miRNA in Rh30- and Rh41-derived exosomes compared to cell lysates, respectively, 155 of which were commonly deregulated in both cell lines (Fig. 2d). There were 31 differentially deregulated miRNA in common among the ERMS (JR1 and RD) and ARMS (Rh30 and Rh41) cell lines (Fig. 2e); 7 were also common with Rh36 cell line.

We next focused on enriched miRNA only, since these may be responsible for the observed paracrine-mediated effects of RMS cells, as they can be delivered to recipient cells. We found that 34 miRNA were commonly enriched in exosomes of the 2 ERMS cell lines JR1 and RD; 2 of these miRNA were also in common with the Rh36 cell line (Fig. 2f). In ARMS-derived exosomes, 62 miRNA were commonly enriched in Rh30 and Rh41 exosomes (Fig. 2g). Finally, there were 10 miRNA in common among the ERMS (JR1 and RD) and ARMS (Rh30 and Rh41) cell lines, and only 2 miRNA (miR-1246 and miR-1268) were commonly enriched in all RMS exosomes including Rh36 (Fig. 2h). By qRT-PCR analysis, we verified enrichment of 7 out of 9 tested miRNA transcripts, using the noncoding small nuclear U6 RNA for normalization (Fig. 2i). RNAse A treatment of exosome isolates prior to miRNA extraction did not alter the level of enrichment of the identified miRNA (Supplementary Fig. S1a). To further investigate whether the identified exosomal miRNA were indeed being taken up by recipient cells and are able to influence the downstream effects in those cells, we evaluated expression levels of 2 experimentally-proven downstream targets of 2 of the identified miRNA: CDK2 (target of miR-638^19^ which is enriched in ARMS exosomes) and CEBPß (target of miR-92b-star^20^ which is enriched in ERMS exosomes). We found that, as expected, protein levels of both were decreased in lysates of recipient cells of these respective exosomes after 48 hours of treatment, when compared to control-treated cells (Fig. 2j).

We analyzed the 62 miRNA commonly enriched in exosomes derived from both ARMS cell lines using the Ingenuity Pathway Analysis (IPA) software. The highest statistically significant experimentally observed and predicted diseases and functions were within the category of ‘Cancer’, followed by ‘Organismal Injury’, ‘Reproductive System disease’, and ‘Inflammatory Disease’ (Table 1). As for the 34 miRNA commonly enriched in the 2 ERMS cell lines JR1 and RD, significantly represented categories included ‘Connective Tissue Disorders’, ‘Inflammatory Disease’, ‘Organismal Injury’ and ‘Cancer’ (Table 2). For the 10 miRNA common among the 4 cell lines (Rh30, Rh41, RD, and JR1), significantly represented categories were predicted to be involved in categories of ‘Cancer’, ‘Organismal Injury’, ‘Reproductive System’, and ‘Inflammatory disease’, due primarily to 2 of the enriched miRNA (Table 3).

Focusing on the 2 miRNA common to exosomes derived from all 5 RMS cell lines (miR-1246 and miR-1268), we employed Target Scan software to identify their putative targets. The top PANTHER pathways identified in which putative targets were involved included Wnt signaling pathway, GnRH receptor pathway, Inflammation, Cadherin and Integrin signaling pathways, Angiogenesis, PDGF, EGF, TGF-beta signaling pathways, along with apoptosis, p53 and Ras pathways, among others (Supplementary Table S1).

**Figure 3:**
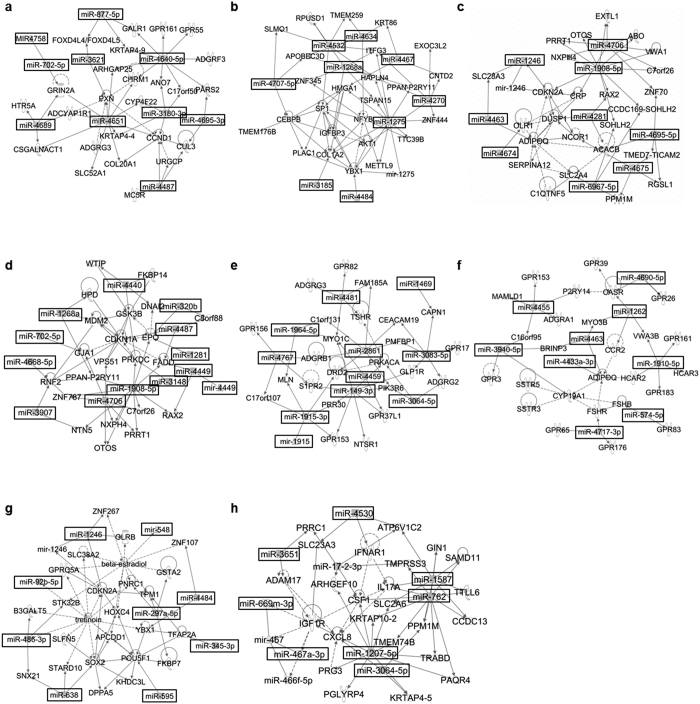
Network-based analysis of miRNA commonly enriched in ERMS- and ARMS-derived exosomes. (**a**–**c**) The top three predicted networks in ERMS-derived exosomes and (**d**–**h**) the top five predicted networks in ARMS-derived exosomes by IPA analysis. Boxes identify miRNA that are commonly enriched in the RMS-derived exosomes in each network. Edges (lines and arrows between nodes) represent direct (solid lines) and indirect (dashed lines) interactions between molecules as supported by information in the Ingenuity knowledge base.

Finally, IPA network analysis showed that, for miRNA enriched in ERMS-derived exosomes, there were 8 significantly represented networks, 3 of which included more than one identified focus miRNA, with nodes centering on proteins implicated in cancer cell cycle biology such as Cyclin D1 (CCND1) (Fig. 3a); IGFBP3, AKT1, SP1, HMGA1, NFYB, and YBX1 (Fig. 3b); and CDKN2A (Fig. 3c). In ARMS cells, miRNA commonly enriched in exosomes showed 12 significant networks, 5 of which included more than one identified miRNA, with nodes centering on proteins implicated in cancer biology, metastasis, and stemness, such as GSK3b, MDM2, CDKN1A (Fig. 3d); PRKACA and MYO1C (Fig. 3e); CASR, CCR2 and BRINP3 (Fig. 3f); CDKN2A, SOX2, YBX1 and POU5F1 (Fig. 3g); and CXCL8, IFNAR1, CSF1, and IGF1R (Fig. 3h).

### RMS-derived exosomes enhance proliferation of recipient human fibroblasts and recipient RMS cells

To test whether exosomes have functional paracrine effects on recipient cells, we then assessed the proliferation of normal human BJ fibroblasts as well as RMS cells after treatment with RMS-derived exosomes. For these studies, and to identify a possible dose-dependent effect, we used 1X and 10X concentrations of exosomes, such that 1X refers to an amount derived from a number of RMS cells equivalent to the number of recipient cells, while 10X is ten times that amount. Of note, the amount of protein in 1X and 10X concentrations was on average 0.25 μg/ml and 2.5 μg/ml, respectively. These amounts are both within the lower ranges of tumor exosome quantities used in published studies, which seem to range from 0.5 to 40 μg/ml, when reported^6^,^13^.

**Figure 4:**
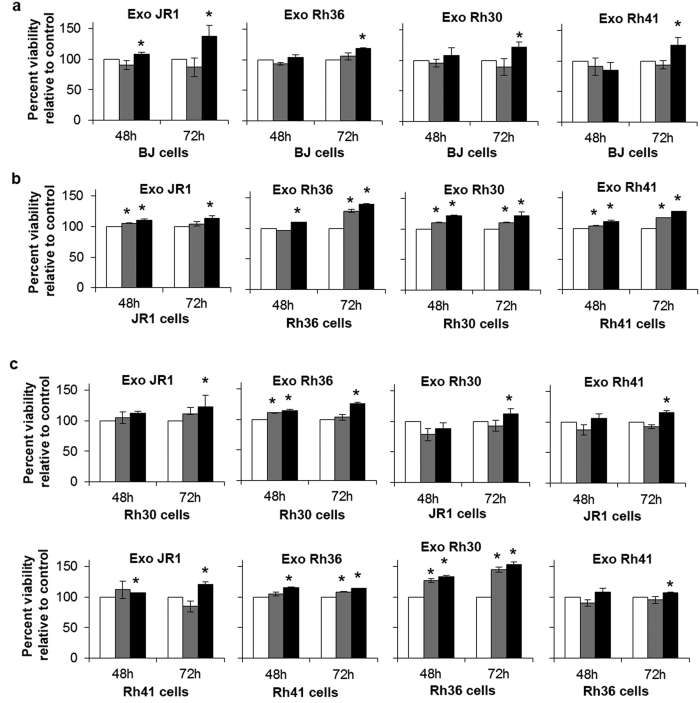
RMS-derived exosomes induce the proliferation of normal fibroblasts and tumor cells. (**a**) Percent viability by MTT assay of normal BJ fibroblasts treated with RMS-derived exosomes (Exo) from the specified cell lines, normalized to that of cells treated with control exosome-free media. (**b**) Percent viability by MTT assay of the specified RMS cells treated with their own respective RMS-derived exosomes (Exo), normalized to that of cells treated with control exosome-free media. (**c**) Percent viability by MTT assay of the specified RMS cells treated with the specified RMS-derived exosomes (Exo), normalized to that of cells treated with control exosome-free media. Cells were treated for 48 and 72 hours (h), as specified. Conditions shown include cells treated with exosome-free media (control, white bars), 1X exosomes (grey bars) and 10X exosomes (black bars), as specified. Values represent means of at least 3 independent experiments, each performed in triplicate. Bars represent standard deviations. Asterisks represent significant p-value <0.05 compared to control condition.

We used exosomes derived from 2 ERMS cell lines: Rh36 and JR1, and 2 ARMS cell lines: Rh30 and Rh41. Treatment of human BJ fibroblasts with exosomes derived from the ERMS or the ARMS cell lines did not increase proliferation at the 1X concentration, but an effect became evident after 72 hours at the 10X concentration (Fig. 4a). Treatment of each RMS cell line with its own exosomes at the 1X concentration enhanced the proliferation of the parental cells at 48 hours for all cell lines except Rh36, and at 72 hours for all cells except JR1, while treatment with 10X exosomes had a significant effect at both 48 and 72 hours in all cell lines (Fig. 4b). We then evaluated whether exosomes derived from ARMS cells would influence proliferation of ERMS cells, and *vice versa*. In all cases, exosome treatment at 10X resulted in an increase in proliferation of the recipient cells by 72 hours, and in some cases effects were also seen at 1X concentration and at 48 hours of treatment (Fig. 4c). From the above data, we conclude that RMS-derived exosomes can increase the proliferation of recipient fibroblasts, ERMS, and ARMS cells, in a seemingly dose-dependent manner. The observed effects on fibroblast proliferation were also verified using another human fibroblast cell line, the IMR90 fetal lung fibroblasts (Supplementary Fig. S2a).

### RMS-derived exosomes induce migration and invasion of normal fibroblasts, and promote angiogenesis

**Figure 5:**
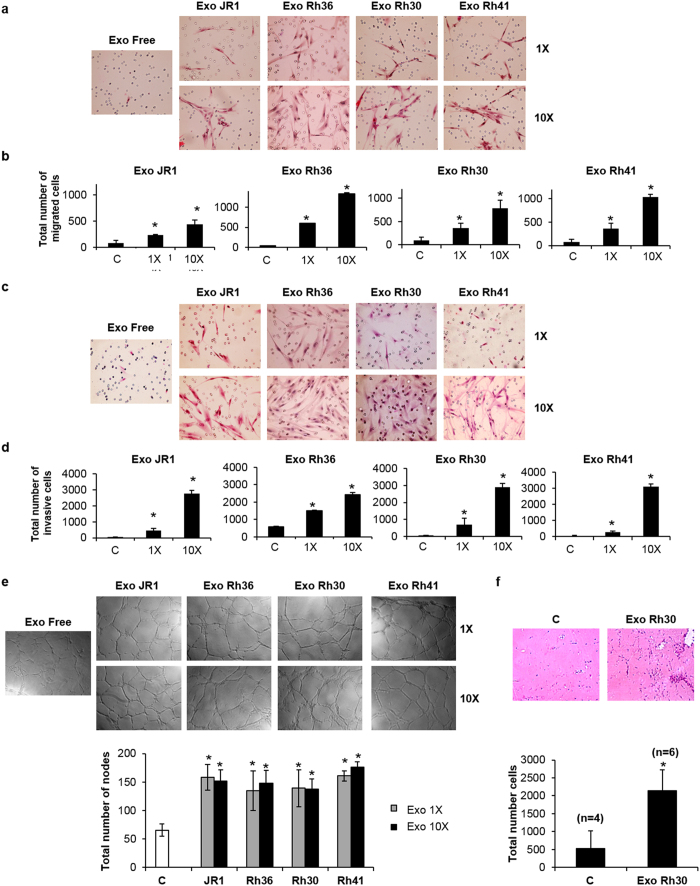
RMS-derived exosomes induce the migration and invasion of normal BJ fibroblasts and promote angiogenesis. Representative photomicrographs for (**a**) transwell migration assay and (**c**) matrigel invasion assay, of normal BJ fibroblasts after treatment with exosomes from the specified cell lines, at 1X and 10X exosome concentration. The control condition is fibroblasts treated with exosome-free (Exo Free) medium. (**b**) and (**d**) Quantitation of the total number of migrated cells in (**a**) and invading cells in (**c**), respectively, at the denoted conditions of exosome-free media (control C), 1X and 10X exosome concentrations. (**e**) Representative phase contrast photomicrographs of endothelial tube formation of HUVECs cultured on Matrigel with 1X or 10X RMS-derived exosomes from the specified cell lines, as well as control (Exo Free medium), as specified. Quantitation of the total number of nodes in the conditions shown. Grey bars represent the exosome 1X concentration, and black bars the 10X concentration, as specified. All histograms represent the means of at least 3 independent experiments, each performed in triplicate. Bars represent standard deviations. Asterisks represent significant p-value <0.05, compared to control condition. (**f**) Representative light microscopy photomicrographs of H&E stained growth factor–reduced Matrigel plugs containing either PBS (as a vehicle control, denoted C) or Rh30-derived exosomes (Exo Rh30), harvested 7 days after subcutaneous injection into NOD/SCID mice. Quantitation of the total number of cells within the matrigel plug. Histograms represent means for 4 plugs from control ‘C’ PBS-loaded matrigel, and 6 plugs from exosome-loaded matrigel ‘Exo Rh30’. Bars represent standard deviations. Asterisks denote p-value <0.05 compared to control.

We next investigated the effect of RMS-derived exosomes on invasive and migratory properties of normal fibroblasts, as tumor-associated fibroblasts have been shown to play a primary role in local invasion and metastasis in solid tumors (reviewed in ref. 21). At both 1X and 10X concentrations, treatment with ERMS-derived or ARMS-derived exosomes resulted in a clear and significant increase in migration of normal BJ fibroblasts, in a seemingly dose-dependent fashion (Fig. 5a,b). Similarly, ERMS- and ARMS-derived exosomes both resulted in a significant increase in fibroblast invasion through matrigel at both the 1X and 10X concentrations, and the effects were more prominent with increased exosome concentration (Fig. 5c,d). Similar effects were observed when using a second human fibroblast cell line, the IMR90 fetal lung fibroblasts (Supplementary Fig. S2b–e). There was no change in the observed effect on fibroblast migration and invasion when exosome isolates were first treated with RNAse A and DNAse (Supplementary Fig. S1b–e).

Next, to investigate the effect of RMS-derived exosomes on angiogenesis, we conducted a matrigel tube formation assay that evaluates the ability of human umbilical vascular endothelial cells (HUVECs) to differentiate into capillary-like structures when plated on matrigel^22^. Addition of RMS-derived exosomes, at both the 1X and 10X concentrations, resulted in a similar increased cellularity of the network formation compared to cells treated with exosome-free medium (Fig. 5e, upper panel). Counting the nodes which are defined as the intersection of 3 or more branches of tubular connections showed a similar greater than 2-fold increase in the number of interconnecting HUVECs when treated with either 1X or 10X RMS-derived exosomes compared to controls (Fig. 5e, lower panel). To evaluate the effects of exosomes on cell migration and invasion *in vivo*, we performed a matrigel plug assay, where matrigel plugs were loaded with 100 μg of Rh30-derived exosomes, or equivalent amount of diluent (phosphate-buffered saline), injected subcutaneously into the abdomen of immunosuppressed mice and collected 7 days later^23^,^24^. We found that exosome-loaded matrigel plugs had significantly increased number of infiltrating host cells compared to the control (Fig. 5f), consistent with the results obtained *in vitro*.

## Discussion

Despite multiple attempts at intensifying chemotherapeutic approaches to treatment, limited improvements in survival have been made for patients with advanced stage RMS over the past 10 years^25^. This underlies the need to better understand the biology of this tumor in order to identify novel targets for therapy. Since exosomes have been shown to be important in mediating metastasis in other types of cancer such as melanoma, breast, and pancreatic cancer^10^,^26^,^27^,^28^, we hypothesized that they may be also important in RMS-mediated paracrine signaling and invasion. We found that RMS cell lines, whether of embryonal or alveolar histologies, secrete quantifiable amounts of exosomes. In fact, when we tried to isolate exosomes from normal human fibroblasts (BJ cells) or normal human skeletal myoblasts (SkMC cells), the number of exosomes isolated was too low to quantify or characterize (negative data not shown). This is in keeping with other studies which suggest that cancer cells may secrete higher quantities of exosomes compared to normal cells^17^,^29^. Increased levels of exosomes are also detected in body fluids of cancer patients when compared to those from healthy controls^17^, and similar results are noted in sera of xenograft-bearing mice^29^. Notably, all 5 RMS cell lines that we tested were found to secrete similar quantities of exosomes, as quantified by protein content.

Cancer cell-derived exosomes are implicated in tumor growth, survival, angiogenesis, escape from immune surveillance, and stimulating migration and invasion (reviewed in ref. 14). Importantly, tumor-derived exosomes have been reported to be both necessary and sufficient for preparing the pre-metastatic niche and supporting tumor cell metastasis, by stimulating angiogenesis, modulating stromal cells and remodeling extracellular matrix^10^,^26^,^30^, and organotropism of specific metastases was recently found to be dictated by paracrine signaling through exosomes^27^,^31^. Relatively few studies have evaluated the role of exosomes in paracrine signaling of mesenchymal tumors such as sarcomas, or in pediatric cancers. Our study now shows that exosomes derived from both ERMS and ARMS cells exert a positive effect on recipient tumor and normal fibroblast cell proliferation. Notably, we found that RMS-derived exosomes had a prominent effect on inducing migration and invasion of normal human BJ and IMR90 fibroblasts, as well as on stimulating angiogenesis of endothelial cells in culture. This shows that both RMS histologies known to be locally invasive can influence non-cancerous supporting cells in the microenvironment *via* exosomes, to facilitate local invasion and possibly metastasis. *In vivo*, we identified a role for RMS-derived exosomes in facilitating host cell infiltration into matrigel plugs, which again supports a role in promoting invasion and angiogenesis during RMS tumor progression. As with all exosome isolation techniques, residual amounts of extravesicular protein complexes, RNA, or DNA influencing the observed phenotype cannot be completely excluded. Even though exosome treatment with RNAse A and DNAse did not diminish the effects on fibroblast cell migration and invasion, this does not completely rule out the possibility of such extra-exosomal molecules contributing to the observed results.

The PAX3/7-FOXO1 fusion oncoprotein is thought to be the initiating tumor promoting insult in fusion-positive rhabdomyosarcoma. Fusion-negative and fusion-positive RMS are distinct tumor subtypes, with different pathways of oncogenic deregulation, and likely divergent cells of origin (reviewed in ref. 1). Interestingly, we found that miRNA content of fusion-positive ARMS-derived exosomes clustered together even to a higher extent than did the respective cellular miRNA, suggesting common specific paracrine mediators in this highly metastatic RMS subtype. Importantly, most of these miRNA were not shared with the fusion-negative ERMS-derived exosomes. Of note, the Rh36 cell line - which is the only ERMS cell line in our panel with wild-type p53 - had very few miRNA differentially enriched in exosomes, and this was notably different from the remainder of the examined cell lines. While there is some emerging evidence that p53 may play a role in modulating exosome content^32^,^33^, whether the p53 status of Rh36 cells contributes to this observation is yet unclear and needs to be further investigated. This variance seems to also be consistent with studies in different cell lines and cell types, where some studies showed that the miRNA profile of exosomes reflects that of the parent cells^34^, while other studies found that miRNA can be selectively enriched or depleted from exosomes, suggesting that their incorporation into exosomes occurs by a selective sorting mechanism^6^. Notably, even when excluding the Rh36 cell line, we found only 10 miRNA commonly enriched between the ERMS (JR1 and RD) and ARMS (Rh30 and Rh41) exosomes, further demonstrating the difference in underlying biology of these distinct tumor subtypes.

Several studies have attempted to identify panels of exosome-derived miRNA that can be used as biomarkers in different types of cancer^17^,^18^. The identified exosome miRNA in our study may therefore be used as a starting point, with specific subsets of differentially enriched miRNA as candidate biomarkers of fusion-positive and fusion-negative RMS, respectively. However, our study evaluated only miRNA that were significantly enriched in exosomes compared to respective cell lysates, since we were primarily interested in miRNA that may have specific effects on paracrine signaling and therefore would be expected to be selectively packaged into exosomes. Studies to identify biomarkers may need to include miRNA present at relatively high levels in exosomes, irrespective of levels present in the respective cells. Such studies would optimally employ RNA sequencing technologies, and would be expected to identify a larger number of exosome-expressed miRNA.

Our analysis showed that exosomes derived from the 2 ERMS (fusion-negative) cell lines JR1 and RD, had commonly enriched miRNA implicated in cancer-related disease categories such as connective tissue disorders, inflammation, and cancer. Significantly represented networks featured proteins previously implicated in ERMS biology such as Cyclin D1, IGF, AKT, SP1, and CDKN2A^35^,^36^,^37^,^38^,^39^. Network analysis also suggested target proteins that, though not previously studied in RMS, have been implicated in other tumor types with roles in cell proliferation such as NFYB^40^, invasion and metastasis such as YBX1^41^, and cellular transformation such as HMGA1^42^. The findings of protein nodes previously implicated in ERMS biology supports the role of exosomes as mediators of such pathway alterations, while the findings of novel cancer-associated protein networks opens possibilities for investigation of such pathways in ERMS tumors.

As for fusion-positive ARMS cell lines, IPA analysis revealed that commonly enriched exosome miRNA had experimentally observed and predicted diseases and functions within the categories of cancer, inflammation, connective tissue diseases, and angiogenesis, all of which suggest a role in tissue invasion. Significantly represented networks featured proteins previously implicated in ARMS biology (GSK3b, MDM2, CDKN1A, CDKN2A, IGF1R)^43^,^44^,^45^,^46^,^47^, proteins involved in immune evasion and tumor metastasis (CXCL8, CSF1, IFNAR1, CCR2, CASR)^48^,^49^,^50^,^51^,^52^, stemness (SOX2, POU5F1)^53^,^54^, tumor cell proliferation and invasion (YBX1)^41^, or have been associated with other tumor types with a possible role in tumor biology (PRKACA, MYO1C, BRINP3)^55^,^56^,^57^. The finding of the miRNA networks targeting proteins involved in stemness, immune evasion and metastasis, enriched in ARMS but not ERMS exosomes, suggests that paracrine signaling via exosomes may have differential effects on mediating the known aggressive behavior of ARMS. Further studies will now focus on validation of these candidate proteins in human samples and cell lines, and investigation of validated pathways as therapeutic targets.

Interestingly, there were only 2 miRNA that were commonly enriched among all 5 RMS cell lines, namely miR-1246 and miR-1268. The miRNA miR-1246 has been reported as a possible biomarker in esophageal squamous cell carcinoma^58^, colorectal cancer^59^ and cervical cancer^60^, and its antagonism led to suppression of proliferation, colony formation and invasion in non-small cell lung cancer cell lines^61^. Conversely, very little is known regarding the functions of miR-1268, other than that it is expressed in gestational diabetes mellitus^62^, and may be implicated in facioscapulmohumeral muscular dystrophy^63^. Using the Target Scan software and PANTHER analysis, we identified 4395 putative targets of miR-1246 and miR-1268 combined, with effects on pathways implicated in tumorigenesis, such as Wnt, Cadherin, EGF and FGF, angiogenesis, and apoptosis signaling. Interestingly, the top 2 pathways identified (namely Wnt and GnRH signaling pathways) were among the top 15 statistically significant pathways implicated as targets of miRNA upregulated in exosomes secreted by murine C2C12 myoblasts during differentiation^64^. This may reflect the importance of deregulation of differentiation pathways during tumorigenesis, knowing that RMS cells have an activated myogenic program and likely originate from myoblast precursor cells (reviewed in ref. 1). The identification of these common miRNA among both ERMS and ARMS-derived exosomes is therefore particularly interesting, as it may offer a common therapeutic target for both disease subtypes. Further studies using functional assays to specifically investigate the contribution of these identified miRNA to RMS tumor biology are currently underway.

The observed effects of RMS-derived exosomes on cell proliferation, migration, invasion, and angiogenesis may be due to multiple exosome components, including miRNA, but also protein, mRNA, and even DNA fragments, all of which have been identified in exosomes of different cell lines and likely contribute to paracrine signaling^5^. For example, differentially expressed exosome proteins such as integrins can direct their ‘homing’ to specific organs to enhance the metastatic process^31^. Further characterization of RMS exosome protein, mRNA, and other molecular content in addition to the miRNA will be needed to better understand the mechanism by which these particles exert their physiologic effects, and to identify which of these components are in fact responsible for the observed effects on proliferation, migration, invasion, and metastasis.

## Methods

### Cell culture

The human ERMS cell lines JR1 and Rh36, and the ARMS cell lines Rh30 and Rh41, were generously donated by Dr. Peter Houghton (Columbus, OH, USA), and have been previously described (reviewed in ref. 65). RD (human ERMS), BJ (human foreskin fibroblast), IMR90 (human fetal lung fibroblast), HUVEC (human umbilical vein endothelial cells) and HEK293T (human embryonic kidney) cell lines were purchased from ATCC (Manassas, VA). Mouse embryonic fibroblasts (MEFs) were isolated from E13.5 embryos of wild type mice of a mixed C57BL/6 × 129/Sv 77 genetic background (Jackson Laboratory, Maine). All RMS cells were tested and authenticated by DDC Medical (London, UK). Cells were cultured in Dulbecco’s Modified Eagles Medium or RPMI-1640 medium supplemented with 10% fetal bovine serum, 1% glutamine, and 1% Pen/Strep (all from Sigma), maintained under standard incubator conditions (humidified atmosphere, 95% air, 5% CO_2,_ 37 °C) and passaged twice weekly by trypsinization.

### Exosome isolation and PKH26 staining

Exosome-free medium was prepared by ultracentrifugation of medium supplemented with 20% FBS, at 100,000 × g overnight at 4 °C to effectively deplete exosomes^66^. The resulting supernatant was filtered with 0.22 μm filter (Millipore), and diluted 1:1 in FBS-free medium to reach 10% FBS concentration. Cells were incubated in 150 mm plates in exosome-free medium for 72 hours, conditioned media were collected and exosomes isolated by differential centrifugation as per ‘Basic Protocol 1’ described by Théry *et al.*^67^. The pellet was resuspended directly in PBS for functional assays, in lysis buffer for protein extraction, in Trizol^®^ (Invitrogen) for miRNA extraction, or in 200 μg/ml RNAse A with or without 200 μg/ml DNAse (Roche) for 30 min at 37 °C. For all functional assays, cells were treated with exosomes at 1X and 10X concentration, where 1X corresponds to the quantity of exosomes isolated from a known number of cells and used to treat equivalent number of cells and 10X exosomes corresponds to 10 times the 1X quantity. For PKH26 (Sigma) staining, exosomes were isolated with the following changes: following the first 100,000 × g centrifugation, the pellet was resuspended in 500 μL PKH26 membrane dye-diluent C (Sigma) and incubated for 5 minutes at room temperature, along with a control aliquot containing PKH26 membrane dye-diluent C without exosomes. The labeling reaction was stopped by adding an equal volume of 1% BSA. Labeled exosomes were ultracentrifuged at 100,000 × g for 70 minutes, and the pellet was resuspended in medium.

### GFP transduction, confocal and scanning electron microscopy

Retrovirus expressing GFP under control of MSCV promoter (MSCV-GFP) was generated using calcium phosphate transfection of HEK293T packaging cells (ATCC), then titrated. Virus was then added with 8 μg/ml Polybrene (hexadimethrine bromide, Sigma) to cultured MEFs, followed by spinoculation at 32 °C and 2500 rpm for 2 hours. Medium was replaced after 3 hours, and process was repeated the next day. GFP-transduced MEFs were then exposed to PKH26-stained exosomes or control diluent for 2 hours. For live-cell imaging, cells were plated on a confocal dish (MatTek corporation, USA). Images were acquired at 37 °C every 15 minutes for 2 hours, using a Zeiss LSM 710 confocal microscope (Zeiss, Germany) and a 63X objective lens. Z-stacks were acquired every 0.2 μm for the complete depth of the cells. For Scanning Electron Microscopy (SEM), exosome pellets were fixed in 2% paraformaldehyde and 1% glutaraldehyde (Sigma). The sample was then applied to a continuous carbon grid, washed 8 times in distilled H_2_O, then dehydrated with graded ethanol steps, left to dry, and observed using a Zeiss SEM at 30 kV. ImageJ software (http://rsbweb.nih.gov/ij/) was used to quantify the diameter of the photographed exosomes.

### Protein analysis

Cells and exosomes were lysed using CHAPS lysis buffer (30 mM Tris-Cl, pH 7.5; 150 mM NaCl; 1% CHAPS) mixed with 25X protease inhibitor (Roche), and sonication. Proteins were quantified by Bradford method (Bio-Rad). Electrophoresis was performed using 10% acrylamide gel, proteins were transferred to polyvinylidene difluoride (PVDF) membranes (Bio-Rad) and probed with the following antibodies: anti-TSG101 (Abcam), anti-HSC70, anti-GAPDH, anti-Calnexin, anti-C/EBPβ, anti-CDK2 (all from Santa Cruz Biotechnology), anti-FOXO-1 (Cell Signaling), and appropriate species-specific HRP-conjugated secondary antibodies (Santa Cruz), and detected using ECL reagent (Roche).

### RNA extraction, miRNA profiling and analysis

Total RNA was isolated from exosome samples and respective cells using Trizol^®^ (Invitrogen) according to manufacturer’s instructions. RNA concentrations were determined by absorption at 260-nm wavelength with an ND-1000 spectrometer (Nanodrop Technologies, Wilmington, DE, USA). The Experion electrophoresis system using the standard RNA chips (Bio-Rad) was used to assess RNA quality and generate electropherograms. Expression microarrays were performed using Affymetrix GeneChip miRNA 3.0 Arrays kit as described by the manufacturer (Affymetrix, Santa Clara, CA, USA). The arrays were washed and stained on the Affymetrix Fluidics station 450, scanned with an Affymetrix gene chip scanner 3000 7 G (Affymetrix, Santa Clara, CA, USA) and analysed with the R statistical environment. Data normalization was performed using *rma* algorithm^68^, annotations were derived using *biomaRt*^69^, differential expression measured using *LIMMA*^70^ and visualization for differentially expressed miRNAs using *gplots*. We used a 1.5-fold change and a false discovery rate (FDR) < 0.05 as the cut-off level. The identified miRNA were analysed using Ingenuity Pathway Analysis (IPA) software (Ingenuity^®^ Systems, www.ingenuity.com), to identify diseases, functions, and networks in which the miRNA identified are implicated. Fisher’s exact test was used to assign statistical significance (p < 0.05), and each network’s score was displayed as −log (p value). Target Scan version 7.0 (http://www.targetscan.org) was used to predict gene targets of the 2 common deregulated miRNA identified, and Panther Software (http://pantherdb.org) was used to identify the biological pathways linked to these targets.

### Quantitative real-time polymerase chain reaction

The TaqMan miRNA Assays (Applied Biosystems) hsa-miR-1246, hsa-miR-4726-5p, hsa-miR-92b-5p, hsa-miR-4689,hsa-miR-4487, hsa-miR-4793-3p, hsa-miR-595, has-miR-3148 and hsa-miR-486-3p were used, and the noncoding small nuclear U6 RNA (RNU6) was used as endogenous control. Reactions were performed in triplicate in 10 μl volumes, on at least 3 different biologic replicates. Quantitative miRNA data were acquired and analysed using the CFX96 real-time PCR detection system (Bio-Rad). Ct values >35 were considered to be below the detection level of the assay. Therefore, only the miRNA with a Ct ≤ 35 were included in the analyses. Relative miRNA expression levels were compared via the 2−ΔΔCt method^71^.

### MTT cell viability assay

Cells were seeded in 96-well plates and cultured as above. The following day, medium was replaced by exosome-free medium and treated with exosomes at 1X and 10X concentration, as specified. Cells incubated in exosome-free medium were used as a control. MTT cell viability assay (Roche) was performed according to the manufacturer’s instructions. Results were computed as the mean percent absorbance of exosome-treated condition relative to control (exosome-free condition). Each experiment was repeated at least 3 times, each performed in triplicate.

### Migration, invasion, and tube formation assays

BD Falcon™ Cell Culture Inserts with 8 μm pore size were used for migration assay and the same inserts coated with 10% growth factor reduced Matrigel were used for invasion assay (BD Biosciences). Cells were seeded onto the top chamber of a transwell insert in 300 μl exosome-free medium and 600 μl of serum-free medium was added into the bottom chamber in a 24-well plate. Exosomes were added four hours later onto the top chamber, at 2 concentrations as detailed above (1X and 10X). Cells incubated in exosome-depleted medium were used as a control. The inserts were fixed after 24–72 hours, stained with hematoxylin and eosin (H&E stain), mounted and coverslipped. Slides were air-dried, sealed, photographed and the migrating/invading cells counted using ImageJ software.

The formation of capillary-like structures was assessed in a 96-well plate using growth factor-reduced Matrigel (BD Biosciences). HUVECs (1.5 × 10^4 ^cells/well) were plated on top of Matrigel in endothelial basal medium containing 1% FBS. Exosomes were added at a concentration of 1X and 10X, and exosome-free medium was used as control. Cells were incubated for 2 hours and then evaluated by phase-contrast microscopy and photographed. The number of nodes (defined as the intersection of 3 or more branches) formed in each condition was counted using the ImageJ software.

### Matrigel plug *in vivo* assay

Animal experiments were conducted in accordance with the American University of Beirut Institutional Animal Care and Use Committee guidelines; all animal studies were approved by this committee. Six week-old NOD/SCID mice (NOD. CB17-*Prkdc*^scid^, Jackson Laboratory) were injected subcutaneously along the abdominal midline with 500 μL growth factor-reduced Matrigel. Prior to the injection, Rh30-derived exosomes (in PBS) corresponding to 100 μg of protein were added to the Matrigel. An equal volume of PBS was used in the Matrigel control. Mice were sacrificed one week later, Matrigel plugs were removed, fixed in 4% formaldehyde solution, and embedded in paraffin. Slides were stained with hematoxylin/eosin and photographed using light microscope, and infiltrating cells were counted using ImageJ Software.

### Statistical analysis

Comparisons between experimental groups were performed using Student’s t-test, and a p-value below 0.05 was considered as statistically significant. All data is presented as mean ± standard deviation.

## Additional Information

**How to cite this article**: Ghayad, S. E. *et al.* Exosomes derived from embryonal and alveolar rhabdomyosarcoma carry differential miRNA cargo and promote invasion of recipient fibroblasts. *Sci. Rep.* **6**, 37088; doi: 10.1038/srep37088 (2016).

**Publisher’s note:** Springer Nature remains neutral with regard to jurisdictional claims in published maps and institutional affiliations.

## Supplementary Material

Supplementary Information

## Figures and Tables

**Table 1:** IPA analysis of significantly represented diseases and functions categories for miRNA commonly enriched in ARMS exosomes (Rh30, Rh41).

Category	p-value^a^	Identified miRNA
Cancer	3.4 × 10^−7^–4.7 × 10^−2^	miR-1268, mir-548, miR-595, miR-1915-3p, mir-1915, miR-320b, mir-550, miR-195-3p, miR-1207-5p, miR-638, miR-1228-5p
Organismal Injury and Abnormalities	3.4 × 10^−7^–4.7 × 10^−2^	miR-1268, miR-1908-5p, mir-548, miR-595, miR-1915-3p, mir-1915, mir-550, miR-320b, miR-195-3p, miR-1207-5p, miR-638, miR-1228-5p
Reproductive System Disease	3.4 × 10^−7^–2.3 × 10^−2^	miR-1268, mir-548, miR-1915-3p, mir-1915, miR-1207-5p, miR-638
Inflammatory Disease	9.7 × 10^−5^	miR-1908-5p, miR-320b, miR-638
Inflammatory Response	9.7 × 10^−5^	miR-1908-5p, miR-320b, miR-638
Renal and Urological Disease	9.7 × 10^−5^	miR-1908-5p,miR-320b, miR-638
Connective Tissue Disorders	2.8 × 10^−3^	miR-1207-5p, miR-149-3p
Hematological Disease	2.8 × 10^−3^	miR-1207-5p, miR-149-3p
Immunological Disease	2.8 × 10^−3^	miR-1207-5p, miR-149-3p
Cardiovascular Disease	8.5 × 10^−3^–4.3 × 10^−2^	miR-320b
Cardiovascular System Development and Function	2.1 × 10^−2^	miR-320b
Cellular Development	2.1 × 10^−2^–4.8 × 10^−2^	miR-320b, mir-467
Cellular Function and Maintenance	2.1 × 10^−2^	miR-320b
Cellular Growth and Proliferation	2.1 × 10^−2^–4.8 × 10^−2^	miR-320b, mir-467
Organismal Development	2.1 × 10^−2^	miR-320b
Tissue Development	2.1 × 10^−2^	miR-320b
Gastrointestinal Disease	2.3 × 10^−2^–4.7 × 10^−2^	mir-548, miR-320b, mir-550
Cellular Movement	3 × 10^−2^	miR-320b
Tissue Morphology	4.3 × 10^−2^	miR-320b
Respiratory Disease	4.7 × 10^−2^	miR-320b
Respiratory System Development and Function	4.8 × 10^−2^	mir-467

^a^p-value is indicated as a range for several lower level categories within each category.

**Table 2:** IPA analysis of significantly represented diseases and functions categories for miRNA commonly enriched in ERMS exosomes (JR1, RD).

Category	p-value^a^	Identified miRNA
Connective Tissue Disorders	3.6 × 10^−4^	miR-4270, miR-1275
Hematological Disease	3.6 × 10^−4^	miR-4270, miR-1275
Immunological Disease	3.6 × 10^−4^	miR-4270, miR-1275
Inflammatory Disease	4.8 × 10^−4^	miR-4651, miR-1908-5p
Inflammatory Response	4.8 × 10^−4^	miR-4651, miR-1908-5p
Organismal Injury and Abnormalities	4.8 × 10^−4^–2.7 × 10^−2^	miR-1268, miR-4651, miR-1908-5p, miR-1228-5p
Renal and Urological Disease	4.8 × 10^−4^	miR-4651, miR-1908-5p
Cancer	8.41 × 10^−3^–2.7 × 10^−2^	miR-1268, miR-1275, miR-1228-5p
Reproductive System Disease	2.7 × 10^−2^	miR-1268

^a^p-value is indicated as a range for several lower level categories within each category.

**Table 3:** IPA analysis of significantly represented diseases and functions categories for miRNA commonly enriched in 4 RMS cell line exosomes (Rh30, Rh41, JR1, RD).

Category	p-value^a^	Identified miRNA
Cancer	9.1 × 10^−3^	miR-1268
Organismal Injury and Abnormalities	9.1 × 10^−3^–1.14 × 10^−2^	miR-1268, miR-1908-5p
Reproductive System Disease	9.1 × 10^−3^	miR-1268
Inflammatory Disease	1.1 × 10^−2^	miR-1908-5p
Inflammatory Response	1.1 × 10^−2^	miR-1908-5p
Renal and Urological Disease	1.1 × 10^−2^	miR-1908-5p

^a^p-value is indicated as a range for several lower level categories within each category.
