# Supplementary material for: Exosomes derived from embryonal and alveolar rhabdomyosarcoma carry differential miRNA cargo and promote invasion of recipient fibroblasts
Source: Sci Rep. 2016 Nov 17;6:37088. doi: 10.1038/srep37088 (PMC5112573; doi:10.1038/srep37088)
Supplement: Supplementary Information [file srep37088-s1.doc]

**Exosomes derived from embryonal and alveolar rhabdomyosarcoma carry differential miRNA cargo and promote invasion of recipient fibroblasts**

Sandra E. Ghayad^*1^, Ghina Rammal^1, 2^, Farah Ghamloush^2^, Hussein Basma^2^, Rihab Nasr^3^, Mona Diab-Assaf^4^, Claude Chelala^5^, Raya Saab^*2, 3^

^1^Department of Biology, Faculty of Science II, EDST, Lebanese University, Fanar, Lebanon.

^2^Department of Pediatric and Adolescent Medicine, American University of Beirut, Beirut, Lebanon.

^3^Department of Anatomy, Cell Biology and Physiology, American University of Beirut, Beirut, Lebanon.

^4^Department of Chemistry and Biochemistry, Faculty of Sciences II, EDST, Lebanese University, Fanar, Lebanon.

^5^Centre for Molecular Oncology, Barts Cancer Institute, Barts and the London School of Medicine and Dentistry, Queen Mary University of London, London, UK.

* **Correspondence to**:

Raya Saab, American University of Beirut, Pediatric Hematology/Oncology, Riad El Solh Street, Beirut 1107 2020, Lebanon. Phone: +961-1-350000, ext. 4780. Fax: +961-1-377384. Email: [rs88@aub.edu.lb](mailto:rs88@aub.edu.lb)

Sandra E. Ghayad, Lebanese University, Faculty of Science II, Department of Biology, Fanar, Jdeidet, P.O. Box 90656, Lebanon. Phone: +961-70-043 128. Fax: +961-1-377384. Email: [sandra.ghayad@ul.edu.lb](mailto:sandra.ghayad@ul.edu.lb)

**Supplementary Table S1. PANTHER pathway analysis for** putative targets of miR-1246 and miR-1268

| **Pathway** | **Number of Proteins** |
| --- | --- |
| Wnt signaling pathway | 80 |
| Gonadotropin releasing hormone receptor pathway | 76 |
| Inflammation mediated by chemokine and cytokine signaling pathway | 61 |
| Cadherin signaling pathway | 52 |
| Integrin signalling pathway | 46 |
| CCKR signaling map | 45 |
| Angiogenesis | 43 |
| Heterotrimeric G-protein signaling pathway-Gi alpha and Gs alpha mediated pathway | 41 |
| PDGF signaling pathway | 40 |
| EGF receptor signaling pathway | 34 |
| Huntington disease | 33 |
| Heterotrimeric G-protein signaling pathway-Gq alpha and Go alpha mediated pathway | 31 |
| FGF signaling pathway | 31 |
| Alzheimer disease-presenilin pathway | 30 |
| TGF-beta signaling pathway | 30 |
| Apoptosis signaling pathway | 28 |
| Endothelin signaling pathway | 24 |
| p53 pathway | 23 |
| Nicotinic acetylcholine receptor signaling pathway | 23 |
| Ubiquitin proteasome pathway | 22 |
| Cytoskeletal regulation by Rho GTPase | 21 |
| Parkinson disease | 20 |
| Interleukin signaling pathway | 19 |
| Ras Pathway | 19 |
| Muscarinic acetylcholine receptor 1 and 3 signaling pathway | 18 |
| Metabotropic glutamate receptor group III pathway | 18 |
| Toll receptor signaling pathway | 17 |
| Muscarinic acetylcholine receptor 2 and 4 signaling pathway | 17 |
| 5HT2 type receptor mediated signaling pathway | 17 |
| Alzheimer disease-amyloid secretase pathway | 16 |
| VEGF signaling pathway | 15 |
| Oxytocin receptor mediated signaling pathway | 14 |
| Angiotensin II-stimulated signaling through G proteins and beta-arrestin | 14 |
| T cell activation | 13 |
| Metabotropic glutamate receptor group I pathway | 13 |
| Insulin/IGF pathway-mitogen activated protein kinase kinase/MAP kinase cascade | 13 |
| B cell activation | 13 |
| Axon guidance mediated by netrin | 12 |
| PI3 kinase pathway | 12 |
| Metabotropic glutamate receptor group II pathway | 12 |
| Thyrotropin-releasing hormone receptor signaling pathway | 12 |
| Blood coagulation | 12 |
| Synaptic_vesicle_trafficking | 11 |
| Ionotropic glutamate receptor pathway | 11 |
| Insulin/IGF pathway-protein kinase B signaling cascade | 11 |
| p53 pathway feedback loops 2 | 11 |
| Histamine H1 receptor mediated signaling pathway | 11 |
| 5HT1 type receptor mediated signaling pathway | 11 |
| Alpha adrenergic receptor signaling pathway | 10 |
| De novo purine biosynthesis | 10 |
| Dopamine receptor mediated signaling pathway | 10 |
| GABA-B_receptor_II_signaling | 9 |
| Heterotrimeric G-protein signaling pathway-rod outer segment phototransduction | 9 |
| Beta1 adrenergic receptor signaling pathway | 9 |
| Axon guidance mediated by Slit/Robo | 8 |
| Transcription regulation by bZIP transcription factor | 8 |
| Interferon-gamma signaling pathway | 8 |
| p38 MAPK pathway | 8 |
| Nicotine pharmacodynamics pathway | 8 |
| Beta2 adrenergic receptor signaling pathway | 8 |
| Adrenaline and noradrenaline biosynthesis | 7 |
| Notch signaling pathway | 7 |
| p53 pathway by glucose deprivation | 7 |
| Enkephalin release | 7 |
| Cortocotropin releasing factor receptor signaling pathway | 7 |
| DNA replication | 7 |
| Oxidative stress response | 6 |
| Hypoxia response via HIF activation | 6 |
| Vasopressin synthesis | 6 |
| Opioid proopiomelanocortin pathway | 6 |
| Hedgehog signaling pathway | 6 |
| General transcription by RNA polymerase I | 6 |
| Endogenous_cannabinoid_signaling | 5 |
| Opioid prodynorphin pathway | 5 |
| Opioid proenkephalin pathway | 5 |
| FAS signaling pathway | 5 |
| O-antigen biosynthesis | 4 |
| Formyltetrahydroformate biosynthesis | 4 |
| Plasminogen activating cascade | 4 |
| Vitamin D metabolism and pathway | 4 |
| Salvage pyrimidine ribonucleotides | 4 |
| Histamine H2 receptor mediated signaling pathway | 4 |
| Pyrimidine Metabolism | 4 |
| Cholesterol biosynthesis | 4 |
| Cell cycle | 4 |
| 5HT4 type receptor mediated signaling pathway | 4 |
| 5HT3 type receptor mediated signaling pathway | 4 |
| Axon guidance mediated by semaphorins | 3 |
| N-acetylglucosamine metabolism | 3 |
| Methylmalonyl pathway | 3 |
| Heme biosynthesis | 3 |
| De novo pyrimidine deoxyribonucleotide biosynthesis | 3 |
| P53 pathway feedback loops 1 | 3 |
| Glycolysis | 3 |
| General transcription regulation | 3 |
| Circadian clock system | 3 |
| Methionine biosynthesis | 2 |
| Mannose metabolism | 2 |
| Fructose galactose metabolism | 2 |
| De novo pyrmidine ribonucleotides biosythesis | 2 |
| Ascorbate degradation | 2 |
| Androgen/estrogene/progesterone biosynthesis | 2 |
| Adenine and hypoxanthine salvage pathway | 2 |
| ATP synthesis | 2 |
| Thiamine metabolism | 2 |
| Salvage pyrimidine deoxyribonucleotides | 2 |
| Purine metabolism | 2 |
| Beta3 adrenergic receptor signaling pathway | 2 |
| Peptidoglycan biosynthesis | 2 |
| Pentose phosphate pathway | 2 |
| 5-Hydroxytryptamine degredation | 2 |
| Methylcitrate cycle | 1 |
| Lysine biosynthesis | 1 |
| mRNA splicing | 1 |
| Glutamine glutamate conversion | 1 |
| Folate biosynthesis | 1 |
| TCA cycle | 1 |
| Cysteine biosynthesis | 1 |
| Asparagine and aspartate biosynthesis | 1 |
| Arginine biosynthesis | 1 |
| JAK/STAT signaling pathway | 1 |
| Aminobutyrate degradation | 1 |
| Xanthine and guanine salvage pathway | 1 |
| Tryptophan biosynthesis | 1 |
| 2-arachidonoylglycerol biosynthesis | 1 |
| Succinate to proprionate conversion | 1 |
| Serine glycine biosynthesis | 1 |
| Nicotine degradation | 1 |
| S adenosyl methionine biosynthesis | 1 |
| Pyruvate metabolism | 1 |
| Gamma-aminobutyric acid synthesis | 1 |
| Proline biosynthesis | 1 |

**Supplementary Figure S1. Enrichment of identified miRNA in exosome extracts is not due to extra-vesicular miRNA, and exosome effects on fibroblast migration and invasion is not influenced by extra-vesicular RNA or DNA.**

**
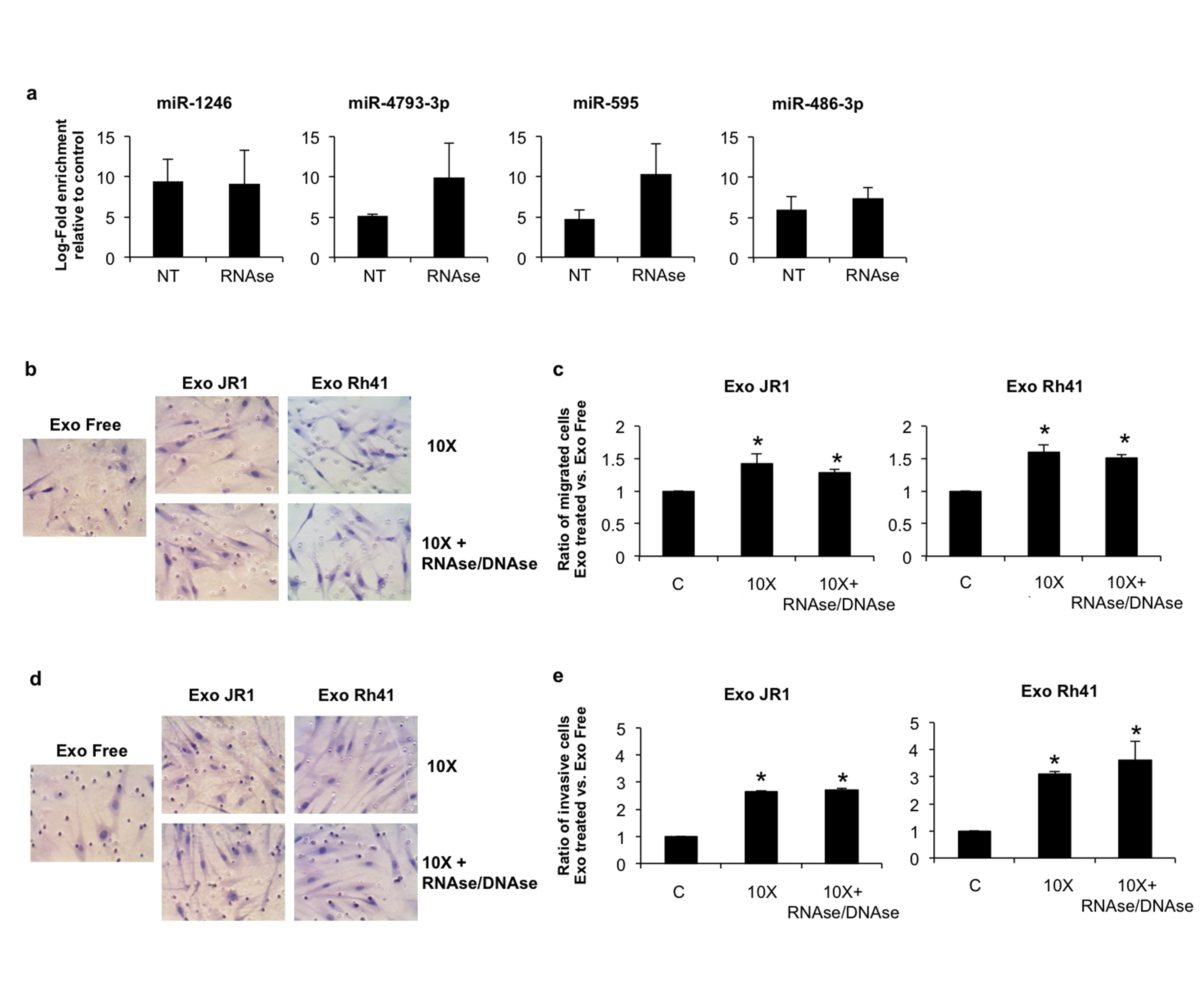
**(**a**) Quantitation by qRT-PCR of the specified enriched miRNA after treatment of Rh41 exosomes with RNAse A, compared to those without RNAse A treatment (NT). The small RNA RNU6 was used as endogenous control. Histograms represent the mean log-fold change of miRNA in exosomes relative to the donor cells, from at least two independent experiments each performed in triplicate. Bars represent standard deviation. Representative photomicrographs for (**b**) transwell migration assay and (**d**) matrigel invasion assay of normal BJ fibroblasts treated with exosomes from the specified cell lines at 10X exosome concentration, and treated with RNAse A/DNAse as indicated. The control condition is fibroblasts treated with exosome-free (Exo Free) medium. (**c**) and (**e**) Quantitation of the number of migrated cells in (b) and invading cells in (d), respectively, relative to control condition ‘C’ of exosome free media, at the denoted conditions of exosome concentrations 10X and 10X + RNAse A/DNAse treated. Histograms represent the means of at least 3 independent experiments. Bars represent standard deviations. Asterisks represent significant p-value < 0.05, compared to control condition.

**Supplementary Figure S2. RMS-derived exosomes induce the proliferation, migration and invasion of IMR90 fibroblasts.**

**
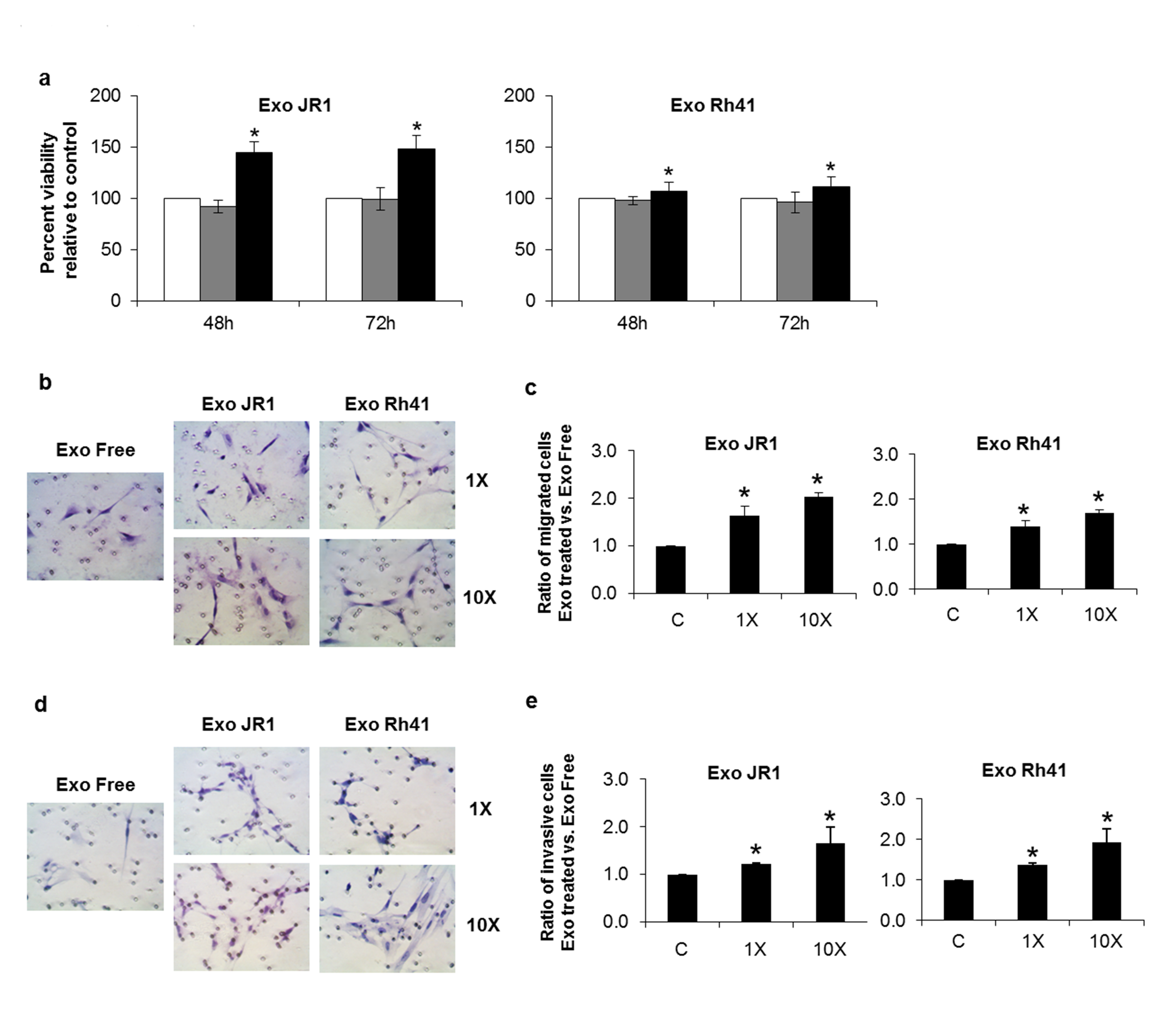
**

(**a**) Percent viability by MTT assay of normal IMR90 fibroblasts treated with RMS-derived exosomes (Exo) from the specified cell lines, normalized to that of cells treated with control exosome-free media, ‘C’. Cells were treated for 48 and 72 hours (h), as specified. Conditions shown include cells treated with exosome-free media (control, white bars), 1X exosomes (grey bars) and 10X exosomes (black bars), as specified. Values represent means of at least 3 independent experiments, each performed in triplicate. Bars represent standard deviations. Asterisks represent significant p-value < 0.05 compared to control condition. Representative photomicrographs for (**b**) transwell migration assay and (**d**) matrigel invasion assay of normal IMR90 fibroblasts treated with exosomes from the specified cell lines, at 1X and 10X exosome concentration, as indicated. The control condition is fibroblasts treated with exosome-free (Exo Free) medium. (**c**) and (**e**) Quantitation of the number of migrated cells in (b) and invading cells in (d), respectively, relative to control condition ‘C’ of exosome free media, at the denoted conditions of 1X and 10X exosome concentrations. Histograms represent the means of at least 3 independent experiments. Bars represent standard deviations. Asterisks represent significant p-value < 0.05, compared to control condition.
